# Looking like the locals - gut microbiome changes post-release in an endangered species

**DOI:** 10.1186/s42523-019-0012-4

**Published:** 2019-10-03

**Authors:** Rowena Chong, Catherine E. Grueber, Samantha Fox, Phil Wise, Vanessa R. Barrs, Carolyn J. Hogg, Katherine Belov

**Affiliations:** 1 School of Life and Environmental Sciences, University of Sydney, Sydney, NSW 2006 Australia; 2 San Diego Zoo Global, PO Box 120551, San Diego, CA 92112 USA; 3 Department of Primary Industries, Parks, Water and Environment, Hobart, TAS Australia; 4 Toledo Zoo, 2605 Broadway, Toledo, OH 43609 USA; 5 Marie Bashir Institute for Infectious Diseases and Biosecurity, Sydney Medical School, University of Sydney, Sydney, NSW 2006 Australia; 6 Sydney School of Veterinary Science, University of Sydney, Sydney, NSW 2006 Australia

**Keywords:** Captivity, Carnivore, Reintroduction program, Wildlife translocation, Dysbiosis

## Abstract

**Background:**

Captivity presents extreme lifestyle changes relative to the wild, and evidence of microbiome dysbiosis in captive animals is growing. The gut microbiome plays a crucial role in host health. Whilst captive breeding and subsequent reintroduction to the wild is important for conservation, such efforts often have limited success. Post-release monitoring is essential for assessing translocation success, but changes to the microbiome of released individuals are poorly understood. The Tasmanian devil was previously shown to exhibit loss of microbiome diversity as a result of intense captive management. This current study examines changes in the devil gut microbiome in response to translocation and aims to determine if perturbations from captivity are permanent or reversible.

**Methods:**

Using 16S rRNA amplicon sequencing, we conducted temporal monitoring of the gut microbiome of released devils during two translocation events, captive-to-wild and wild-to-wild. To investigate whether the microbiome of the released devils changed following translocation, we characterized their microbiome at multiple time points during the translocation process over the course of 6–12 months and compared them to the microbiome of wild incumbent devils (resident wild-born devils at the respective release sites).

**Results:**

We showed that the pre-release microbiome was significantly different to the microbiome of wild incumbent animals, but that the microbiomes of animals post-release (as early as 3 to 4 weeks post-release) were similar to wild incumbents. The gut microbiome of released animals showed significant compositional shifts toward the wild incumbent microbiome of both translocation events.

**Conclusion:**

Our results suggest that the devil gut microbiome is dynamic and that loss of microbiome diversity in captivity can be restored following release to the wild. We recommend the broader application of microbiome monitoring in wildlife translocation programs to assess the impacts of translocation on animal microbiomes.

**Electronic supplementary material:**

The online version of this article (10.1186/s42523-019-0012-4) contains supplementary material, which is available to authorized users.

## Background

Captive breeding and releases are commonly practiced to support global conservation efforts for endangered/threatened species [1–3], such as the California condor (*Gymnogyps californianus*) [4], Arabian oryx (*Oryx leucoryx)* [5] and black-footed ferret *(Mustela nigripes*) [6]. In theory, the release of captive bred animals back into their natural habitats can help re-establish local extinct populations, or supplement declining populations in the wild. In practice however, reintroducing wildlife species from captivity to the wild is often met with very little success [7]. Despite efforts by zoos and captive institutions to replicate an animal’s natural habitat, extreme lifestyle changes in diet, environment and social structures, as well as veterinary interventions, are inevitable in the artificial settings of captivity. In turn, such lifestyle changes incurred in captivity can have multiple flow-on effects on the host-associated microbiome.

The study of the microbiome in wildlife species is still relatively new and has potential to improve conservation efforts. The microbiome, particularly the gut microbiome, has been shown to play an integral role in supporting host health, nutrition, immune functions and even behavior [8–12]. Disturbances to the typical gut microbiome, or dysbiosis, can potentiate the onset or development of various diseases, including diabetes [13, 14], inflammatory bowel disease [15, 16], obesity [13, 17], susceptibility to infections [18] and impaired immunity [8, 10]. There is growing evidence that captivity alters the microbiome, with many species across diverse taxa showing signals of perturbation and microbial diversity loss in captivity compared to their wild counterparts (e.g. [19–22]). While the exact consequences of microbiome perturbations on host health and survival remain poorly understood, these findings have implications for the way captive populations and reintroduction programs are managed. It is conceivable that an altered or depauperate microbiome may have adverse impacts, and so impair their host survival following release to the wild, especially if they are depleted of beneficial microbes. For example, the gut microbiome of captive black howler monkeys (*Alouatta pigra*) lacks microbes that produce butyrate (such as *Butyrivibrio* spp.) in their gut microbiome, which is thought to provide energy for mammalian colon cells and has important health benefits [23]. In the grouse (*Tetrao urogallus*), anatomical changes in the gut, such as shorter small intestines and caeca [24], as well as microbiome disturbances [25] have been observed in captive individuals [25]. These changes can compromise digestion and nutrient absorption and may explain the high mortality of captive birds upon reintroduction to the wild [24–26]. Importantly, different species vary in the way their microbiome responds to changing environments. For example, primates and carnivorous species tend to be more susceptible to microbiome alterations in captivity compared to herbivorous species, whose microbiome generally remains stable [21, 22, 27, 28].

Another species that experiences microbiome perturbations in captivity is the Tasmanian devil (*Sarcophilus harrisii,* hereafter ‘devil’) [22]. The devil is the world’s largest carnivorous marsupial, found only in the wild on the island state of Tasmania, Australia. It is now listed as endangered due to a transmissible cancer, devil facial tumour disease (DFT1 and DFT2) [29, 30] that has decimated most wild populations by up to 80% since its first discovery in 1996 [31]. Conservation actions by the Save the Tasmanian Devil Program (STDP) have included the establishment of an insurance metapopulation in 2006 [32], the introduction of devils to another nearby island (Maria Island) in 2012 and 2013 [33], and the reintroduction of devils back into wild sites on mainland Tasmania between 2015 and 2018 [34]. The primary goal of the insurance metapopulation is to maintain a genetically robust and healthy population of devils that will act as a source of animals for supplementing wild populations under threat. There are currently over 700 devils in the insurance metapopulation, held in a variety of housing types from intensive zoo-based captive facilities (intensive captive, housing 1 to 3 individuals per enclosure) to free-range enclosures (housing 10 to 20 individuals per 10 to 22 ha enclosure) to more wild type settings with a fenced peninsula (30,000 ha) and an island site on Maria Island (9600 ha) [32]. All sites are managed to some extent under the metapopulation framework. The population on Maria Island is home to approximately 100 free-roaming devils and is harvested annually as a source of wild-born devils for reintroduction purposes [35]. (See Additional file 1: Table S1 for detailed site descriptions).

The gut microbiome of the devil was first characterized by Cheng et al. [22] in 2015 and captive devils were shown to have significantly lower microbiome diversity than wild devils. Interestingly, between the two captive facility types, devils housed in intensive captive facilities had lower diversity than devils housed in the free-range enclosures. Although the exact effects of a depauperate microbiome on devils remain largely unknown, a possible consequence is the associated increased risk of obesity, as has been demonstrated in rodent models [36], which could in turn lead to reduced breeding success [22, 37]. As the insurance metapopulation is a source of disease-free devils for reintroductions, it is important to understand how the microbiome will respond to changes in the environment during translocations, whether they be captive-to-wild or wild-to-wild.

In this study, we conducted the first temporal monitoring of the gut microbiome of an endangered carnivorous species throughout two translocations events. Doing so will help us better understand the temporal dynamics of the host-associated gut microbiome, particularly in response to translocation. We aimed to address: i) does the microbiome change over time after the host is translocated, and if so, how? and ii) do released individuals reacquire a “wild-type” microbiome after translocation?

## Methods

### Study populations, translocation events and sample collection

In this study, we focused on two translocation events: captive-to-wild translocation (from intensive captive to Maria Island, 2017, *N* = 8), and wild-to-wild translocation (from Maria Island to Stony Head, 2016, *N* = 17). During both translocations, devils were removed from their respective source locations (intensive captive facilities or Maria Island) and housed in free-range enclosures (10–22 ha) for a period of 6 to 12 weeks prior to their final releases. Whilst in the free-range enclosures, devils were fed a controlled diet consisting of whole carcasses of marsupials including wallaby, pademelon and brushtail possum [38]. (See Additional file 1: Table S1 for detailed site descriptions).

**Fig. 1:**
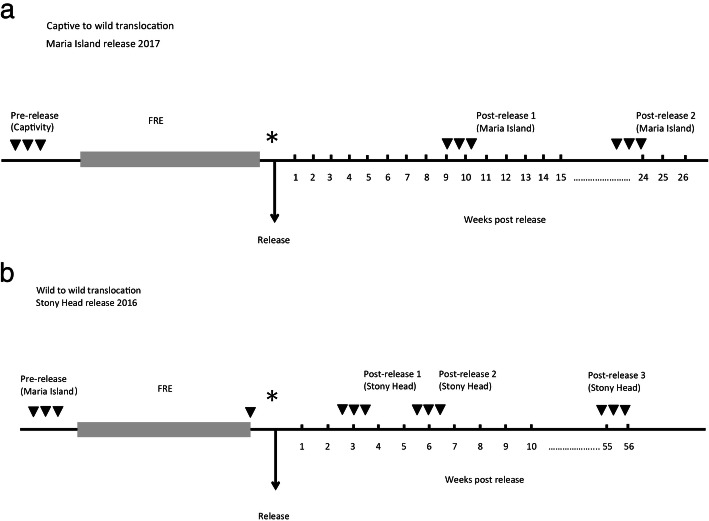
Timeline of the two translocation events. **a** Captive-to-wild translocation (Maria Island release 2017). A total of 8 female devils from the captive insurance population were released. Devils were kept in a free-range enclosure (FRE) for approximately 6 weeks prior to the release. **b** Wild-to-wild translocation (Stony Head release 2016). Seventeen wild devils originating from Maria Island were translocated to Stony Head. Release devils were housed in a free-range enclosure (FRE) for 12 weeks prior to the release. All faecal samples were collected opportunistically at various time points throughout the translocation processes. Arrows represent time points at which faecal samples were collected

Pre-release faecal samples were collected at the source locations by either the keeping or STDP field staff. Faecal samples were also collected at the end of the free-range enclosures period for the wild-to-wild translocation. Post-release trapping and monitoring of the release animals for routine health checks was conducted across two to three time points over a period of 8–12 weeks post- release (See Fig. 1 for translocation timeline and sampling points for the two translocations, and Additional file 2: Table S2 for list of released animals with their respective source locations and samples collected). Thereafter, each release site (Maria Island and Stony Head) was monitored annually through live trapping and camera traps in order to assess the health status of both the release and wild incumbent devils (the term “wild incumbent” hereafter refers to resident wild-born devils that inhabit the respective release site). PVC-pipe traps baited with wallaby meat were set at the release site and surrounding areas [39]. Traps were checked daily between 06:00 and 16:00. As devils are nocturnal, captured devils spent no more than 12 to 22 h inside the traps before being released. Captured devils were identified by their unique microchip numbers and faecal samples were collected opportunistically. All faecal samples were collected from the traps, or hessian sacks used during processing of the animals. As all traps were cleaned after each animal, we can be certain of which sample belonged to which animal. To prevent sample degradation, all faecal samples were collected within 22 h of defecation. Upon collection in sterile vials, all samples were immediately stored at − 20 °C in the field before transferring to the lab where they were stored at − 80 °C until DNA extraction. Faecal samples were also collected from wild incumbent devils captured during all trapping trips following the same procedures as described above. The PVC pipe traps were cleaned and disinfected between each trapping trip using a quaternary ammonium/biguanide veterinary disinfectant (F10SC, Health and Hygiene (Pty) Ltd) to ensure that there was no cross contamination between individuals.

### DNA extraction, sequencing and analysis

To account for contamination, blank negative controls consisted of PCR grade water were used at the DNA extraction, PCR and library preparation and sequencing steps. A sub-portion of sample taken from the core of each faecal sample was used for microbial genomic DNA extraction using the QIAamp DNA Stool Mini Kit (Qiagen).

Thereafter, barcoded amplicons of the 16S ribosomal RNA (rRNA) gene V3-V4 region using the 341F (5′-CCTAYGGGRBGCASCAG-3′) and 806R (5′-GGACTACNNGGGTATCTAAT-3′) primers were generated. Briefly, PCR reactions consisted of a mix of 12.5 μL KAPA HiFi HotStart ReadyMix (Kapa Biosystems), 0.5 μM of each primer and 1 μL of DNA template were used. Thermal cycling conditions were as follows: initial denaturation at 95 °C for 3 min, followed by 35 cycles of denaturation at 95 °C for 30 s, annealing at 55 °C for 30 s, extension at 72 °C for 30 s and a final extension step at 72 °C for 5 min. PCR products were subsequently normalized and pooled using SequalPrep™ Normalization Plate Kit (ThermoFisher) and libraries were purified using Axygen® AxyPrep™ Mag PCR Clean-Up Kit (Fisher Biotec) following the manufacturers’ instructions. Sequencing was performed on a MiSeq (*Illumina*) platform using a MiSeq Reagent kit v3 with a 2 × 300 bp run format at the Ramaciotti Centre for Genomics.

Sequences data was processed and analysed using the QIIME2 (v2019.4) pipeline [40]. Demultiplexed paired-end sequence reads were merged, quality filtered and denoised into amplicon sequence variants (ASVs) using the DADA2 plugin [41]. Low abundance reads were also filtered out to remove potential contaminants in the dataset.

Resulting feature tables were rarified using the minimum number of sequences per sample for diversity analysis; i.e. 3838 sequences for the captive-to-wild translocation and 5530 sequences for the wild-to-wild translocation. Taxonomy was assigned to the ASVs by aligning to the latest Silva 16S database (version 132). Alpha diversity was estimated using the diversity metrics Chao1, number of ASVs and Shannon’s diversity, and statistical significance was assessed using the Kruskal-Wallis tests. Beta diversity was estimated using the UniFrac metrics (weighted and unweighted) and visualized by principle coordinate analysis (PCoA). PERMANOVA tests based on 999 permutations were used to test for differences in microbial beta diversity across sample groups (i.e. Pre-release, Post-release and wild incumbent microbiome). Differential abundance testing was performed using ANCOM implemented in QIIME2 [42].

## Results

Samples were processed and sequenced in two runs. (Run 1 consisted of samples from the wild-to-wild translocation and run 2 consisted of samples from the captive-to-wild translocation.). Sequencing resulted in a total of 10,616,471 paired end reads (6,454,253 paired end reads in run 1 and 4,162,218 paired end reads in run 2). The number of sequences per sample ranges between 107,548 and 327,052 for run 1 and 31,448 to 206,240 for run 2.

### Captive-to-wild translocation (Maria Island release)

Eight captive devils were translocated to Maria Island. A total of 19 faecal samples were collected from five of the eight captive released devils. Faecal samples were also collected from seven wild incumbent devils trapped on the Island, resulting in a total of 26 faecal samples for this translocation (See Additional file 2: Table S2 for list of samples collected, their source locations and collection date). Repeated samples from the same individuals collected at the same time point (due to being trapped more than once during the same trapping trip) were pooled for sequencing. After merging paired-end reads, trimming and quality filtering, a total of 298,942 sequences remained, with a mean of 11,500 sequences per sample.

### Overview of the gut microbiome

**Fig. 2:**
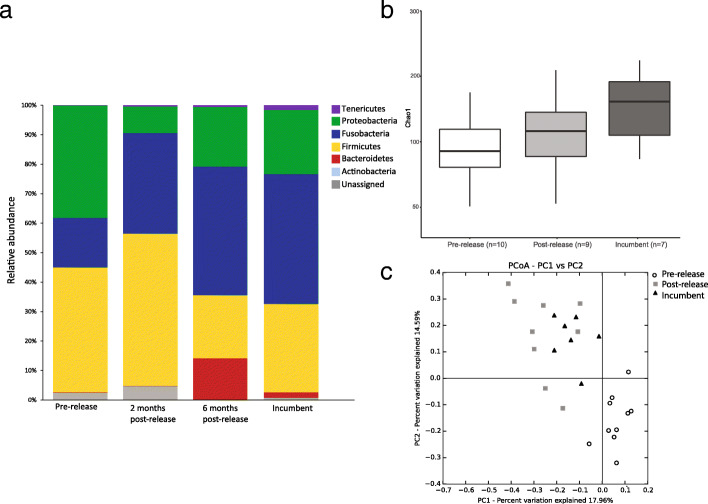
Composition and diversity of gut microbiome of released (*n* = 5) and wild incumbent devils (*n* = 7) from the captive-to-wild translocation (Maria Island release) at various time points. **a** Bar chart showing the phylum level composition of the gut microbiome; **b** Whisker-box plot showing the gut microbiome alpha diversity (*Chao 1*) of released (*n* = 5) and wild incumbent devils (*n* = 7). Black horizontal lines are the median values; the lower and upper bound of boxes represent the 25th and 75th percentiles, respectively; **c** Principal coordinates analysis (PCoA) plot based on the unweighted UniFrac distance matrix, depicting patterns of beta diversity from the gut microbiome of release devils before and after release (pre- and post-release), and the wild incumbent devils on Maria Island (incumbent). Points that cluster together on the ordination have bacterial communities that are more similar

The most dominant bacterial phyla detected across all samples collected from the Maria Island release were Firmicutes (32.84% ± 23.45% SD), Fusobacteria (32.44% ± 23.35% SD), Proteobacteria (21.40% ± 20.87% SD), Bacteroidetes (4.04% ± 9.31% SD), Actinobacteria (0.03% ± 0.08% SD) and Tenericutes (0.65% ± 1.07% SD). Consistent with results from the initial microbiome characterisation [22], the microbiomes of these samples were dominated by the bacterial phylum Firmicutes and showed a high Firmicutes-to- Bacteroidetes ratio (Fig. 2a). Under the Firmicutes phylum, the dominant family detected was *Clostridiaceae* (18.7% ± 19.3% SD). The most dominant members of Proteobacteria detected were of the *Enterobacteriaceae* family. (See Additional file 3: Table S3 for taxonomic composition of all microbiome samples from the captive-to-wild translocation).

### Temporal changes in the microbiome

As expected, the microbiome of Maria Island wild incumbent devils had the highest phylotype richness (alpha diversity by *Chao1,* number of ASVs and Shannon’s diversity), followed by the microbiome of the captive release devils at post-release and pre-release (Kruskal Wallis *p* < 0.05) (Fig. 2b). To examine whether the gut bacterial communities of devils changed significantly after release, we compared the beta diversity (unweighted UniFrac) between pre-release and post-release samples. The average unweighted distance was 0.562 ± 0.009. A PERMANOVA test based on 999 permutations indicated a significant difference (*p* = 0.001) between pre- and post-release samples, with pseudo-F value of 2.751. Conversely, beta diversity (unweighted Unifrac) between wild incumbent and post-release devils (at either two- or six-months post-release) did not differ significantly (*p* = 0.550, pseudo-F value of 0.927), with an average distance of 0.502 ± 0.01. PCoA plot of unweighted UniFrac (Fig. 2c) shows distinct separation between the pre-release microbiome and both post-release and wild incumbent microbiome*,* indicating significant compositional changes after the release. Overlapping of wild incumbent and post-release microbiomes indicates a shift in the captive release microbiome towards the wild incumbent microbiome within the first 6 months following the release.

ANCOM analysis revealed five taxonomic features (ASVs) that were significantly different in abundance between groups (See Additional file 4: Table S4 for ANCOM analysis of differential abundance for the captive-to-wild translocation). ASVs corresponding to the genera *Mycoplasma* (phylum Tenericutes), *Bacteroides* (phylum Bacteroidetes) and *Fusobacterium* (phylum Fusobacteria) were significantly more abundant in both post-release and wild incumbent devils compared to devils before the release (pre-release). ASVs corresponding to various families or genera in Proteobacteria, such as *Enterobacteriaceae* and *Acinetobacter*, and Order Bacillales were most abundant in the pre-release samples.

### Wild-to-wild translocation (stony head release)

Seventeen Maria Island-originated devils were translocated to Stony Head. A total of 41 faecal samples were collected, including 36 samples from nine released devils and five from five wild incumbent devils (See Additional file 2: Table S2 for list of samples collected, their source locations and collection date). Repeated samples from the same individuals collected at the same time point were pooled for sequencing. After merging paired-end reads, trimming and quality filtering, a total of 1,138,280 sequences remained, with a mean of 27,101 sequences per sample.

### Overview of the gut microbiome

**Fig. 3:**
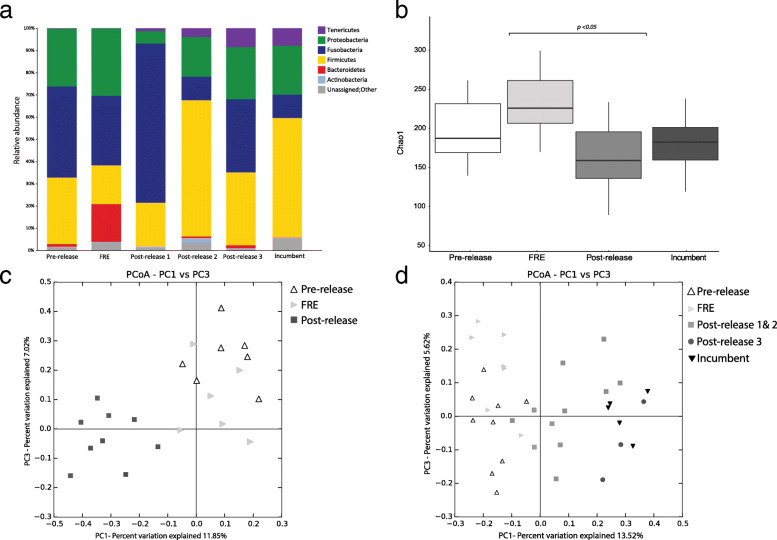
Composition and diversity of gut microbiome of released (*n* = 9) and wild incumbent devils (*n* = 5) from the wild-to-wild translocation (Stony Head release) at various time points. **a** Bar chart showing the phylum-level composition of the gut microbiome; **b** Whisker-box plot showing the gut microbiome alpha diversity (*Chao 1*) of released devils at each time point during translocation. Black horizontal lines are the median values; the lower and upper bound of boxes represent the 25th and 75th percentiles, respectively. Kruskal-Wallis test was used to calculate *p* values and evaluate significance of differences in alpha diversity; **c** Principal coordinates analysis (PCoA) plot based on the unweighted UniFrac distance matrix, depicting patterns of beta diversity from the gut microbial communities of Stony Head released devils at pre-release, the end of the free-range enclosure (FRE) period and post- release (early post-release; 1–3 months post-release). The PCoA plot shows clustering of the pre-release and FRE microbiome, as well as their separation from the post-release microbiome; **d** Principal coordinates analysis (PCoA) plot based on the unweighted UniFrac distance matrix depicting patterns of beta diversity from the gut microbial communities of released (Pre-release, FRE and Post-release 1 & 2 and Post-release 3), and wild incumbent devils at Stony Head (Incumbent). Post-release 1 & 2 represents samples collected at 1 to 3 months post-release, and post-release 3 represents samples collected 1 year post-release. Points that cluster together on the ordination have bacterial communities that are more similar

The most dominant phyla across all samples were Firmicutes (35.20% ± 21.78% SD), Fusobacteria (33.20% ± 23.79% SD), Proteobacteria (21.40% ± 16.48% SD), Bacteroidetes (3.90% ± 8.62% SD), Tenericutes (2.70% ± 5.51% SD) and Actinobacteria (0.70% ± 1.23% SD) (Fig. 3a). Within Firmicutes, the most common bacterial genus was *Clostridium* (14.6% ± 11.22% SD). Bacteria from Fusobacteria were mostly from the genera *Fusobacterium* (23.7% ± 22.36% SD) and *Cetobacterium* (6.7% ± 9.59% SD), while Proteobacteria was dominated by *Plesiomonas* (10.8% ± 11.27% SD) (See Additional file 5: Table S5 for taxonomic composition of all microbiome samples from the wild-to-wild translocation).

### Temporal changes in the microbiome

Initial analysis comparing between the pre-release, free-range enclosures and post-release 1 & 2 (1 month and 3 months post-release respectively) microbiome of the nine Maria Island originated devils showed that phylotype richness (alpha diversity) was the lowest in the post-release samples, with significant differences in alpha diversity found between free-range enclosures and post-release (both post-release 1 and 2) samples (Kruskal-Wallis, *p* < 0.05) (Fig. 3b). Beta diversity analysis (unweighted Unifrac) revealed significant differences between post-release samples (post-release 1 and 2) and both pre-release and free-range enclosures samples (PERMANOVA, *p* = 0.001), with an average unweighted distance of 0.45 ± 0.003 between post-release and pre-release, and 0.51 ± 0.009 between post-release and free-range enclosures. Principle coordinates analysis (PCoA) of the unweighted UniFrac distance matrix shows that post-release samples are separated from both pre-release and free-range enclosures samples, which form a cluster together (Fig. 3c). Principal coordinates PC1 and PC2 together explained 18.87% of the variation between individuals.

Three additional post-release samples collected approximately 1 year after the release in 2017 (Post-release 3) were included in subsequent analysis. Contrary to our expectations, the microbial diversity in the gut microbiome of the released devils did not increase following the release, as phylotype richness (alpha diversity) was the lowest in all the post-release samples. Beta diversity measured by both unweighted and weighted UniFrac again revealed significant differences between samples from different time points (PERMANOVA, *p* < 0.05). Unweighted UniFrac PCoA plot shows clustering of pre-release and free-range enclosures samples, while early post-release samples (post-release 1 & 2) show a shift away from this cluster and towards the wild incumbent microbiome. The three microbiome samples collected 1-year post-release (post-release 3) overlap with both the early post-release (post-release 1 & 2) and wild incumbent samples, suggesting some degree of stability following the initial shift shortly after the release (Fig. 3d).

ANCOM analysis detected three features (ASVs) with significantly different relative abundance between time points. ASV matching to an unclassified genus within the *Bacteroidaceae* family and *Edwardsiella* spp. were significantly more abundant in the free-range enclosures samples compared to samples collected at all other time points, while an ASV matching to *Epulopiscium* spp. was in higher abundance in the pre-release group. (See Additional file 6: Table S6 for ANCOM analysis of differential abundance for the wild-to-wild translocation).

## Discussion

Intensive captive management has altered the devil microbiome, resulting in loss of diversity, particularly in the gut [22]. This observation has prompted us to investigate whether such microbiome perturbations are permanent or can be reversed once the hosts are released to the wild. Here we examined gut microbiome samples collected from released devils over the course of two translocations; i) captive-to-wild translocation (captivity to Maria Island), and wild-to-wild translocation (Maria Island to Stony Head). Our results showed that the devil gut microbiome is not static as there were significant diversity and compositional changes across time for both translocations. Increased microbial diversity was observed in the captive devils released onto Maria Island. Compositional shifts towards the wild incumbent microbiome occurred within 6 to 12 months of post-release in both translocations, suggesting that the loss of microbiome diversity and perturbations previously observed are not permanent.

Changes in gut microbiome from captive-to-wild are similar to a previous study where a captive dugong (*Dugong dugon*) was released into the wild and subsequently recaptured 8 months later. It was found to have a hindgut microbiome similar to those of wild subadult and adult dugongs [43]. The microbiome shift was attributed to exposure to natural conditions and foraging (from cos lettuce fed in captivity to seagrass in the wild), as well as interactions with wild dugongs. Similar factors associated with changes in diets and microbial exposures through environmental and social contacts may also be driving the post-release changes observed in the released devils in our current study. Unlike the dugong study, which provided a snapshot of the post-release microbiome at a single time point, we investigated the temporal variations of the devil gut microbiome across multiple time points throughout the translocation process. Doing so has allowed us to understand the long-term temporal dynamics of the devil gut microbiome in greater detail. Of note, short and long term temporal fluctuations in the gut microbiome of mammals living in the wild are not uncommon, as seen in field mice [44], and red squirrel [45].

Interestingly the wild-to-wild translocation (Stony Head release) results do not reflect an increase in microbiome diversity post-release. At this site, there was a loss of microbial diversity post-release, regardless of the number of months that had passed since the release occurred. This can be potentially attributed to the origin of the devils used in the Stony Head translocation. Although not native to the island, devils living on Maria Island are free roaming and are considered wild [33]. When comparing the wild incumbent microbiome of Stony Head devils to Maria Island devils, the Maria Island animals have a higher microbial diversity, although this is not statistically significant (Kruskal-Wallis test, *p* > 0.05). This higher microbiome diversity may reflect a wider range of food sources available on Maria Island, including wallabies, insects, birds and other marine species such as penguins, shearwater and fish. Stony Head on the other hand is a more homogeneous landscape, being an inland site occupied by mostly farmland and a military base. Potential food sources at this location may not be as varied as Maria Island hence reducing microbiome diversity. Nevertheless, the microbiome of released devils at post-release became more similar to the wild incumbent microbiome, specifically in relation to the acquisition of Tenericutes and Actinobaceteria, both of which were previously missing or in much lower abundance in the pre-release microbiome (Fig. 3a). However, we also found that some members of the microbiome, such as Bacteroidetes, persisted at post-release despite not being present in the wild incumbent microbiome (Fig. 3a). This wild-to-wild translocation therefore shows that at post-release, the microbiome of released devils gained some novel microbes and shifted towards the wild incumbent microbiome, while still maintaining certain microbes that are not found within the wild incumbent community. In addition, we only found a limited number of microbial features (no more than five) that were in significantly different abundances between groups for both translocations. The persistent occurrence and abundance of most bacteria therefore likely represent the devil core gut microbiome. Furthermore, our small sample size may have also contributed to the low number of features with differential abundance across groups.

The general shift in the post-release microbiome towards the wild incumbent microbiome observed here suggests some level of host-host and host-environment microbiome sharing is occurring, in line with similar observations from previous studies in domestic dogs (*Canis lupus familiaris*) [46] and komodo dragons (*Varanus komodoensis*) [47]. This demonstrates that the devil gut microbiome is dynamic and subject to changes that are likely driven by environmental factors, such as diet. For example, higher proportions of Mycoplasma and Tenericutes in both the wild incumbent and post-release devil microbiome on Maria Island compared to the pre-release microbiome of the captive release devils are likely due to increased access and consumption of fish and other marine species on the island. Mycoplasma bacteria has been commonly observed in many marine species including fish and molluscs [48–51]. Members of this phyla often dominate the gut microbiome of fish, with Tenericutes contributing to 18 and 82% of the sequences retrieved from the guts of two king mackerel specimens [52]. Indeed, devils on the island are often observed eating fish that have been washed up on the beach (P. Wise, personal communication, June 2018). Furthermore, social interactions between the release and wild incumbent devils during feeding and mating also likely contributed to the observed microbiome sharing and shift towards the wild incumbent microbiome.

Previously, devils have been found to have high prevalence of Firmicutes (53.5 ± 3.9%) and very low prevalence of Bacteroidetes (1.2 ± 0.6%) in their gut compared to most other mammalian species [22]. A high ratio of gut Firmicutues to Bacteroidetes is common in carnivorous mammals [22, 53] and has been linked to improved efficiency in harvesting energy from food. From the wild-to-wild translocation (Stony Head release), we observed a significant increase in the proportion of Bacteroidetes at the end of the free-range enclosures period (23.56% in free-range enclosures vs. < 1.34% in Pre-release and all Post-release). The reason behind this significant increase in Bacteroidetes and hence changes to the F:B ratio at the end of the free-range enclosures period is unclear but may reflect changes in the pattern of food intake by devils whilst being housed in the free-range enclosures. Devils in the wild often gorge up to 40% of their body mass in a single meal and then do not eat for several days due to limited food sources [54]. The need to efficiently harvest and store energy from food is likely lessened under the more relaxed environment of a free-range enclosure, where animal carcasses (e.g. wallabies, possums and pademelons) are put out for the devils at feed stations on a regular (two to three times per week) basis [38].

For all wildlife species, management of host-associated microbiome in captivity is important and management strategies should be implemented to minimise microbiome perturbations in the first place. This can be achieved through provision of more natural and diverse diets, increased microbial reservoirs in housing enclosures and by minimising antibiotic use. Future studies should focus on determining the health consequences, if any, of microbiome perturbations as a result of captivity. There has also been evidence suggesting that long-term managed species with multiple generations in captivity experience greater microbiome changes and may therefore be less capable of reacquiring wild-type microbiome [55, 56]. Understanding the effects of birth origin and the number of generations in captivity on host-associated microbiome could have important implications for the captive management of wildlife species. Furthermore, in light of the significance of the Devil facial tumour disease (DFTD) on wild devil populations, future studies should compare the microbiome between infected and healthy devils to better understand the interplay between DFTD and microbiome health. Given the close link between the microbiome and host immunity, it is possible that immunosuppression due to DFTD infection [57] may cause considerable changes to the microbiome.

## Conclusions

To the best of our knowledge, this is the first study that has systematically investigated the temporal changes of the host gut microbiome during translocations of a wildlife species. We have shown that a carnivorous species’ microbiome perturbations from captivity are not necessarily permanent, and that translocating animals regardless of their source locations, results in acquisition of the wild incumbent, or resident microbiome. We recommend conservation practitioners to incorporate microbiome monitoring as part of their species recovery programs and post-release monitoring to assess the impacts of translocation on animal microbiomes and understand its implication in translocation success.

## Additional files

Additional file 1: **Table S1.** Descriptions of pre-release (source locations), Free-range enclosures (FRE) and post-release (release locations) sites for each translocation. (DOCX 28 kb)

Additional file 2: **Table S2.** List of microbiome samples from each translocation, including animal ID, origin and sample collection dates. (XLSX 10 kb)

Additional file 3: **Table S3.** Taxonomic compositions of all microbiome samples from the captive-to-wild translocation (Maria Island release) at the phylum, class, order, family, genus levels. (XLSX 129 kb)

Additional file 4: **Table S4.** ANCOM analysis of differential abundance for the captive-to-wild translocation. (XLSX 10 kb)

Additional file 5: **Table S5.** Taxonomic compositions of all microbiome samples from the wild-to-wild translocation (Stony Head release) at the phylum, class, order, family, genus levels. (XLSX 233 kb)

Additional file 6: **Table S6.** ANCOM analysis of differential abundance for the wild-to-wild translocation. (XLSX 9 kb)

## Data Availability

All raw sequence reads are available in the NCBI SRA database with accession number SRP194130.

## Abbreviations

DFTD: Devil facial tumour disease
FRE: Free-range enclosures
STDP: Save the Tasmanian Devil program

## References

[1] Mathews F, Orros M, McLaren G, Gelling M, Foster R (2005). Keeping fit on the ark: assessing the suitability of captive-bred animals for release. Biol Conserv.

[2] Fischer J, Lindenmayer DB (2000). An assessment of the published results of animal relocations. Biol Conserv.

[3] Snyder NFR, Derrickson SR, Beissinger SR, Wiley JW, Smith TB, Toone WD, Miller B (1996). Limitations of captive breeding in endangered species recovery. Conserv Biol.

[4] Toone WD, Wallace MP, Olney PJS, Mace GM, Feistner ATC (1994). The extinction in the wild and reintroduction of the California condor (*Gymnogyps californianus*), in creative conservation: interactive management of wild and captive animals.

[5] Spalton JA, Lawerence MW, Brend SA (2009). Arabian oryx reintroduction in Oman: successes and setbacks. Oryx.

[6] Miller B, Biggins D, Hanebury L, Vargas A, Olney PJS, Mace GM, Feistner ATC (1994). Reintroduction of the black-footed ferret (*Mustela nigripes*), in creative conservation: interactive management of wild and captive animals.

[7] Seddon PJ, Armstrong DP, Maloney RF (2007). Developing the science of reintroduction biology. Conserv Biol.

[8] Kinross JM, Darzi AW, Nicholson JK (2011). Gut microbiome-host interactions in health and disease. Genome Med.

[9] Cho I, Blaser MJ (2012). The human microbiome: at the interface of health and disease. Nat Rev Genet.

[10] Kau AL, Ahern PP, Griffin NW, Goodman AL, Gordon JI (2011). Human nutrition, the gut microbiome and the immune system. Nature.

[11] Flint HJ, Scott KP, Louis P, Duncan SH (2012). The role of the gut microbiota in nutrition and health. Nat Rev Gastroenterol Hepatol.

[12] Cryan JF, Dinan TG (2012). Mind-altering microorganisms: the impact of the gut microbiota on brain and behaviour. Nat Rev Neurosci.

[13] Devaraj S, Hemarajata P, Versalovic J (2013). The human gut microbiome and body metabolism: implications for obesity and diabetes. Clin Chem.

[14] Hartstra AV, Bouter KE, Bäckhed F, Nieuwdorp M (2015). Insights into the role of the microbiome in obesity and type 2 diabetes. Diabetes Care.

[15] Frank DN, Robertson CE, Hamm CM, Kpadeh Z, Zhang T, Chen H, Zhu W, Sartor RB, Boedeker EC, Harpaz N (2011). Disease phenotype and genotype are associated with shifts in intestinal-associated microbiota in inflammatory bowel diseases. Inflamm Bowel Dis.

[16] Fava F, Danese S (2011). Intestinal microbiota in inflammatory bowel disease: friend of foe?. World J Gastroenterol: WJG.

[17] DiBaise John K., Zhang Husen, Crowell Michael D., Krajmalnik-Brown Rosa, Decker G. Anton, Rittmann Bruce E. (2008). Gut Microbiota and Its Possible Relationship With Obesity. Mayo Clinic Proceedings.

[18] Honda K, Littman DR (2012). The microbiome in infectious disease and inflammation. Annu Rev Immunol.

[19] Clayton JB, Vangay P, Huang H, Ward T, Hillmann BM, Al-Ghalith GA, Travis DA, Long HT, Van Tuan B, Van Minh V (2016). Captivity humanizes the primate microbiome. Proc Natl Acad Sci.

[20] Kohl KD, Skopec MM, Dearing MD (2014). Captivity results in disparate loss of gut microbial diversity in closely related hosts. Conserv Physiol.

[21] McKenzie VJ, Song SJ, Delsuc F, Prest TL, Oliverio AM, Korpita TM, Alexiev A, Amato KR, Metcalf JL, Kowalewski M (2017). The effects of captivity on the mammalian gut microbiome. Integr Comp Biol.

[22] Cheng Y, Fox S, Pemberton D, Hogg C, Papenfuss AT, Belov K (2015). The Tasmanian devil microbiome—implications for conservation and management. Microbiome.

[23] Amato KR, Yeoman CJ, Kent A, Righini N, Carbonero F, Estrada A, Gaskins HR, Stumpf RM, Yildirim S, Torralba M (2013). Habitat degradation impacts black howler monkey (*Alouatta pigra*) gastrointestinal microbiomes. ISME J.

[24] Liukkonen-Anttila T, Saartoala R, Hissa R (2000). Impact of hand-rearing on morphology and physiology of the capercaillie (Tetrao urogallus). Comp Biochem Physiol A Mol Integr Physiol.

[25] Wienemann T, Schmitt-Wagner D, Meuser K, Segelbacher G, Schink B, Brune A, Berthold P (2011). The bacterial microbiota in the ceca of Capercaillie (*Tetrao urogallus*) differs between wild and captive birds. Syst Appl Microbiol.

[26] Seiler C, Angelstam P, Bergmann H-H (2000). Conservation releases of captive-reared grouse in Europe–what do we know and what do we need. Cahiers d’Ethologie.

[27] Jesús-Laboy D, Kassandra M, Godoy-Vitorino F, Piceno YM, Tom LM, Pantoja-Feliciano IG, Rivera-Rivera MJ, Andersen GL, Domínguez-Bello MG (2012). Comparison of the fecal microbiota in feral and domestic goats. Genes.

[28] Alfano N, Courtiol A, Vielgrader H, Timms P, Roca AL, Greenwood AD (2015). Variation in koala microbiomes within and between individuals: effect of body region and captivity status. Sci Rep.

[29] Loh R, Bergfeld J, Hayes D, O'hara A, Pyecroft S, Raidal S, Sharpe R (2006). The pathology of devil facial tumor disease (DFTD) in Tasmanian devils (Sarcophilus harrisii). Vet Pathol.

[30] Pye RJ, Pemberton D, Tovar C, Tubio JM, Dun KA, Fox S, Darby J, Hayes D, Knowles GW, Kreiss A (2016). A second transmissible cancer in Tasmanian devils. Proc Natl Acad Sci.

[31] Lazenby BT, Tobler MW, Brown WE, Hawkins CE, Hocking GJ, Hume F, Huxtable S, Iles P, Jones ME, Lawrence C (2018). Density trends and demographic signals uncover the long-term impact of transmissible cancer in Tasmanian devils. J Appl Ecol.

[32] Hogg C, Lee A, Hibbard C. Managing a metapopulation: intensive to wild and all the places in between. In: Hoogs SFCJ, Pemberton D, Belov K, editors. Saving the Tasmanian Devil: recovery through science based managment. in press. Melbourne: CSIRO Publishing.

[33] Wise P, Lee D, Peck S, Clarke J, Thalmann S, Hockley J, Schaap D, Pemberton D. The conservation introduction of Tasmanian devils to Maria Island National Park: a response to devil facial tumor disease (DFTD). Glob Re-introduction Perspect. 2016:166–71.

[34] Fox S, Seddon PJ. Wild devil recovery: managing devil in the presence of disease. In: Hoogs SFCJ, Pemberton D, Belov K, editors. Saving the Tasmanian Devil: recovery through science based management. in press. Melbourne: CSIRO publishing. p. 141–8.

[35] Wise P, Peck S, Clarke J, Hogg CJ. Conservation introduction of Tasmanian devils to Maria Island: a managed response to DFTD. In: Hoogs SFCJ, Pemberton D, Belov K, editors. Saving the Tasmanian Devil: recovery through science based management. in press. Melboune: CSIRO Publishing.

[36] Turnbaugh PJ, Ley RE, Mahowald MA, Magrini V, Mardis ER, Gordon JI (2006). An obesity-associated gut microbiome with increased capacity for energy harvest. nature.

[37] Le Chatelier E, Nielsen T, Qin J, Prifti E, Hildebrand F, Falony G, Almeida M, Arumugam M, Batto J-M, Kennedy S (2013). Richness of human gut microbiome correlates with metabolic markers. Nature.

[38] Izzard S, Barnard O, Schaap D. Managing and maintaining wild temperament and behaviours in captivity. In: Hoogs SFCJ, Pemberton D, Belov K, editors. Saving the Tasmanian Devil: recovery through science based management. in press. Melbourne: CSIRO Publishing.

[39] Hawkins CE, Baars C, Hesterman H, Hocking GJ, Jones ME, Lazenby B, Mann D, Mooney N, Pemberton D, Pyecroft S, Restani M, Wiersma J (2006). Emerging disease and population decline of an island endemic, the Tasmanian devil Sarcophilus harrisii. Biol Conserv.

[40] Bolyen E, Rideout JR, Dillon MR, Bokulich NA, Abnet C, Al-Ghalith GA, Alexander H, Alm EJ, Arumugam M, Asnicar F. QIIME 2: Reproducible, interactive, scalable, and extensible microbiome data science. PeerJ Preprints. 2018. No. e27295v1. 10.1038/s41587-019-0209-9 PMC7015180 31341288

[41] Callahan BJ, McMurdie PJ, Rosen MJ, Han AW, Johnson AJA, Holmes SP (2016). DADA2: high-resolution sample inference from Illumina amplicon data. Nat Methods.

[42] Mandal S, Van Treuren W, White RA, Eggesbø M, Knight R, Peddada SD (2015). Analysis of composition of microbiomes: a novel method for studying microbial composition. Microb Ecol Health Dis.

[43] Eigeland KA, Lanyon JM, Trott DJ, Ouwerkerk D, Blanshard W, Milinovich GJ, Gulino L-M, Martinez E, Merson S, Klieve AV (2012). Bacterial community structure in the hindgut of wild and captive dugongs (Dugong dugon). Aquat Mamm.

[44] Maurice CF, Knowles SC, Ladau J, Pollard KS, Fenton A, Pedersen AB, Turnbaugh PJ (2015). Marked seasonal variation in the wild mouse gut microbiota. The ISME journal.

[45] Bobbie CB, Mykytczuk N, Schulte-Hostedde AI. Temporal variation of the microbiome is dependent on body region in a wild mammal (*Tamiasciurus hudsonicus*). FEMS Microbiol Ecol. 2017;93(7). 10.1093/femsec/fix081 28645188

[46] Song SJ, Lauber C, Costello EK, Lozupone CA, Humphrey G, Berg-Lyons D, Caporaso JG, Knights D, Clemente JC, Nakielny S (2013). Cohabiting family members share microbiota with one another and with their dogs. elife.

[47] Hyde ER, Navas-Molina JA, Song SJ, Kueneman JG, Ackermann G, Cardona C, Humphrey G, Boyer D, Weaver T, Mendelson JR (2016). The oral and skin microbiomes of captive komodo dragons are significantly shared with their habitat. Msystems.

[48] Llewellyn MS, McGinnity P, Dionne M, Letourneau J, Thonier F, Carvalho GR, Creer S, Derome N (2015). The biogeography of the Atlantic salmon (*Salmo salar*) gut microbiome. ISME J.

[49] Lowrey Liam, Woodhams Douglas C., Tacchi Luca, Salinas Irene (2015). Topographical Mapping of the Rainbow Trout (Oncorhynchus mykiss) Microbiome Reveals a Diverse Bacterial Community with Antifungal Properties in the Skin. Applied and Environmental Microbiology.

[50] Bano N, deRae Smith A, Bennett W, Vasquez L, Hollibaugh JT (2007). Dominance of mycoplasma in the guts of the Long-jawed Mudsucker, *Gillichthys mirabilis*, from five California salt marshes. Environ Microbiol.

[51] King GM, Judd C, Kuske CR, Smith C (2012). Analysis of stomach and gut microbiomes of the eastern oyster (*Crassostrea virginica*) from coastal Louisiana, USA. PLoS One.

[52] Givens CE, Ransom B, Bano N, Hollibaugh JT (2015). Comparison of the gut microbiomes of 12 bony fish and 3 shark species. Mar Ecol Prog Ser.

[53] Ley RE, Hamady M, Lozupone C, Turnbaugh PJ, Ramey RR, Bircher JS, Schlegel ML, Tucker TA, Schrenzel MD, Knight R, Gordon JI (2008). Evolution of mammals and their gut microbes. Science.

[54] Pemberton D, Renouf D (1993). A field-study of communication and social-behavior of the Tasmanian devil at feeding sites. Aust J Zool.

[55] Webster NS, Cobb RE, Soo R, Anthony SL, Battershill CN, Whalan S, Evans-Illidge E (2011). Bacterial community dynamics in the marine sponge *Rhopaloeides odorabile* under in situ and ex situ cultivation. Mar Biotechnol.

[56] Becker MH, Richards-Zawacki CL, Gratwicke B, Belden LK (2014). The effect of captivity on the cutaneous bacterial community of the critically endangered Panamanian golden frog (*Atelopus zeteki*). Biol Conserv.

[57] Cheng Y, Makara M, Peel E, Fox S, Papenfuss AT, Belov K (2019). Tasmanian devils with contagious cancer exhibit a constricted T-cell repertoire diversity. Commun Biol.

