# Supplementary material for: Looking like the locals - gut microbiome changes post-release in an endangered species
Source: Anim Microbiome. 2019 Oct 3;1:8. doi: 10.1186/s42523-019-0012-4 (PMC7807427; doi:10.1186/s42523-019-0012-4)
Supplement: Supplementary file 1 — Table S1. Descriptions of pre-release (source locations), Free-range enclosures (FRE) and post-release (release locations) sites for each translocation. (DOCX 28 kb) [file 42523_2019_12_MOESM1_ESM.docx]

| **Captive to wild translocation 2017, (n= 8)** | | | | |
| --- | --- | --- | --- | --- |
|  | **Pre-release**  **(Source location)** | | **Free-range enclosures**  **(no available samples)** | **Post-release  (Release location)** |
|  | Intensive captive  Closed population | | Free-range enclosures (FRE)  [Freycinet FRE]  Closed population | Maria Island  Closed population |
| **Number of devils per site** | 30 to 80 individuals per location (4 locations) | | 8 individuals | ~100 individuals |
| **Size of site** | Individual enclosures 50-100 m^2^ | | 22 ha | 9,672 ha |
| **Site descriptions** | - Intensively managed captive facilities (IC; e.g. zoos) - Enclosures typically 50-100 m^2^, housing individuals singly (adults) or in groups (3-5 juveniles) - High density housing with the ability to control individual pairings - Two facilities located in Tasmania, one in Victoria and one in South Australia - All open enclosures with corrugated fencing - Natural substrates and vegetation such as soil, leaf litter, grass, tussocks, shrubs and logs of various heights provided - Enclosures serviced every couple of days to remove faecal material & uneaten food | | - Large enclosure in coastal heathland with small stands of kunzea, black wattle and banksia - Density of 0.4 devils/ha - Common fauna include Bennett’s wallabies (*Macropus rufogriseus*), pademelons (*Thylogale billardierii*), brushtailed possums (*Trichosurus vulpecula*) and common wombats (*Vombatus ursinus*). A range of species of reptiles, frogs, birds and insects are also abundant. | - National park - Offshore island located 12 km off the south-east coast of Tasmanian - Devils not native to the island and were introduced in 2012, 2013 & 2017 - Density of ~0.01 devils/ha - Vegetation types predominately open-forest of *Eucalyptus obliqua* with grassy, shrubby understorey - Old fallen trees, sand dunes and dolerite boulder fields provide suitable denning substrate and habitat for Tasmanian devils - Common fauna include Forester kangaroo (*Macropus giganteus*), Bennett’s wallaby (*Macropus rufogriseus*), Tasmanian pademelon (*Thylogale biliadierii*), brushtail possum (*Trichosurus vulpecula*), common wombat (*Vombatus ursinus*), short-tailed shearwaters (*Puffinus tenuirostis*), Cape Barren geese (*Cereopsis novaehollandiae*) and leather jacket (*Oligoplites saurus*). A range of species of reptiles, frogs, birds and insects are also abundant. |
| **Typical diet** | - Wallaby/kangaroo, rabbit, beef, day old chicks - Whole prey item at least weekly - Suggested feeding regime of fast and gorge feeds | | - Whole carcasses of natural prey items, predominately Bennett’s wallaby, pademelon, and brushtail possum - Two to three feed sites are used per visit, and are rotated at each visit to encourage activity and foraging behaviour, and prevent devils from feeding in only one area. - Entire carcass is fed out including fur, bone, vital organs and intestines. - Devils fed approximately 80% of the total body mass of the devils held in the FRE per week (i.e. 40% twice weekly) | - Some supplementary feeding of macropod carcasses in the first few weeks of post-release around release sites - Various bird and fish species, insects, ringtail possum, brushtail possum, Tasmanian pademelon, red-necked wallaby, common wombat, echidna, Forester kangaroo |
| **Wild to wild translocation 2016, (n= 17)** | | | | |
|  | **Pre-release**  **(source location)** | **Free-range enclosures** | | **Post-release**  **(Release location)** |
|  | Maria Island  Closed population | Free-range enclosures (FRE)  [Freycinet FRE & Tas Zoo FRE]  Closed population | | Stony Head  Open population |
| **Number of devils per site** | ~100 individuals | Freycinet = 22 individuals; Tas Zoo FRE = 11 individuals | | ~ 15 incumbent devils within study area |
| **Size of site** | 9,672 ha | Freycinet = 22 ha; Tas Zoo FRE = 11 ha | | Study area ~ 24,000 ha |
| **Site descriptions** | As above- refer to the post-release site section for “captive to wild translocation” | - Freycinet = large enclosure in coastal heathland with small stands of kunzea, black wattle and banksia - Tas Zoo FRE = largely black Peppermint forest with tea tree, banksia and melaleuca forming a dense understorey; thickets of button and cutting grasses - Density of 1 – 1.1 devil/ha - Common fauna include Bennett’s wallabies (*Macropus rufogriseus*), pademelons (*Thylogale billardierii*), brushtailed possums (*Trichosurus vulpecula*) and common wombats (*Vombatus ursinus*). A range of species of reptiles, frogs, birds and insects are also abundant. | | - Coastal site on northern coast of Tasmania, occupied predominately by farms, a military base and natural bushland - Density pre-release ~0.0006 devils/ha - Devil facial tumor disease present - Common fauna: Bennett’s wallaby (*Macropus rufogriseus*), Tasmanian pademelon (*Thylogale biliadierii*), brushtail possum (*Trichosurus vulpecula*), common wombat (*Vombatus ursinus*), various bird species, introduced. Domestic species, including rabbit (*Oryctolagus cuniculus*), cattle (*Bos taurus*), sheep (*Ovis aries*), cat (*Felis catus*), Dog (*Canis lupus familiaris*), black rat (*Rattus rattus*) |
| **Typical diet** | As above- refer to the post-release site section for “captive to wild translocation” | - Whole carcasses of natural prey items, predominately Bennett’s wallaby, pademelon, and brushtail possum - Two to three feed sites are used per visit, and are rotated at each visit to encourage activity and foraging behaviour, and prevent devils from feeding in only one area. - Entire carcass is fed out including fur, bone, vital organs and intestines. - Devils fed approximately 80% of the total body mass of the devils held in the FRE per week (i.e. 40% twice weekly) | | - Feed stations were set up at the release site for the first few weeks post-release. - Predominately ringtail and brushtail possum, pademelon, wallaby, other smaller vertebrate species, as well as introduced mammals such as rabbit, mice, cattle, sheep etc |
